# Outcome of catheter ablation in the very elderly‐insights from a large matched analysis

**DOI:** 10.1002/clc.23455

**Published:** 2020-08-31

**Authors:** Kevin Willy, Gerrit Frommeyer, Dirk G. Dechering, Kristina Wasmer, Dennis Höwel, Sarah S. Welle, Nils Bögeholz, Christian Ellermann, Julian Wolfes, Benjamin Rath, Patrick R. Leitz, Julia Köbe, Philipp S. Lange, Patrick Müller, Florian Reinke, Lars Eckardt

**Affiliations:** ^1^ Department of Cardiology II–Electrophysiology University Hospital Münster Münster Germany; ^2^ Department of Cardiology University Hospital Oldenburg Oldenburg Germany

**Keywords:** ablation, arrhythmia, electrophysiology, management

## Abstract

**Background:**

Ablation emerged as first line therapy in the treatment of various arrhythmias. Nevertheless, in older patients (pts), decision is often made pro drug treatment as more complications and less benefit are suspected.

**Hypothesis:**

We hypothesized that different kind of ablations can be performed safely regardless of the pts age.

**Methods:**

We enrolled all pts aged >80 years (yrs) who underwent ablation for three different arrhythmias (atrial flutter [AFL], atrioventricular nodal re‐entry tachycardia [AVNRT], ventricular tachycardia [VT]) between August 2002 and December 2018. Procedural data and outcome were compared with matched groups aged 60 to 80 years and 40 to 60 years, respectively. Periprocedural and in‐hospital complications were analyzed.

**Results:**

The analysis included 1191 patients (397 pts per group: 63% AFL, 23% AVNRT, 14% VT) who underwent ablation. Acute success was high in all types of arrhythmias irrespective of age (>80, 60‐80, 40‐60 years: AFL 97%/98%/98%, AVNRT 97%/95%/97%, VT 82%/86%/93%). Rate of periprocedural complications were similar in all groups treated for AFL and AVNRT. For VT ablations significant differences were noted between pts > 80 or 60 to 80 years and those aged 40‐60 years (16.1%/14.3%/3.6%). Most complications were infections and groin haematoma. No strokes, iatrogenic atrioventricular blocks and deaths related to the ablation occurred.

**Conclusion:**

Ablation appears safe in pts > 80 years. Success rates were comparable to matched younger cohorts. A significant difference was observed for VT patients.

## 1 INTRODUCTION

As life expectancy is steadily increasing and quality of life remains high also in the very elderly, the health system is in a constant transition and challenges have changed over time. With increasing age arrhythmias are becoming clinically manifest in a growing number of patients, too. The need and wish for definitive therapy is also growing due to improved ablation techniques and consequently higher success rates. Catheter ablation has evolved as first line therapy for various arrhythmias. This is reflected in the recently updated ESC guidelines for the management of supraventricular tachycardias (SVT).^1^ Of note, recommendations for ablation procedures are not limited to a special group of patients. While guidelines have devoted paragraphs to the specialized treatment of patients with congenital heart disease or during pregnancy, no specific recommendations are made for older patients.^1^

Atrial fibrillation (AF) and atrial flutter (AFL) are very prevalent in the elderly. For typical AFL in particular, cavotricuspid isthmus ablation (CTI) has evolved as the gold standard since the 1990s in most patients as recurrence rate is high and treatment by CTI characterized by very high success rates.^2^, ^3^ Surprisingly, recent evidence found markedly higher complication rates related to CTI than previously reported.^4^ Advanced age was found to be a major risk factor so that the authors postulated that patient selection for CTI should be made particularly careful in the elderly in whom outcome studies on CTI are scarce. In contrast to AFL, atrioventricular nodal re‐entry tachycardia (AVNRT) manifests/presents most often in young and middle‐aged adults. Interestingly, more recent studies showed, that effectiveness of slow‐pathway modulation was greater in younger patients compared to patients >50 years.^5^ In addition, age seems to be a relevant factor in socioeconomic aspects of AVNRT ablation. Farkowski et al^6^ showed that treatment costs were higher with increased patient age.

While SVT require treatment due to debilitating symptoms, ventricular tachyarrhythmias (VT) in the presence of structural heart disease are life‐threatening and/or may result in adequate defibrillator interventions and therefore demand effective therapy. A contemporary meta‐analysis underlined that VT ablation reduces ICD therapies in patients with coronary heart disease.^7^ This is of importance as Sweeney et al^8^ demonstrated an increased mortality in patients receiving ICD shocks. Of note, increasing age was associated with a higher rate of ablation‐related complications and therefore implemented in a risk score predicting complications and in‐hospital mortality on the basis of over 25.000 patients from a national US database.^9^ Besides, Yousuf et al^10^ showed a 1‐year mortality of 15% after VT ablation and a rate of 7.5% of major adverse events as well as high rates of hospitalization for recurrent arrhythmias or heart failure.

In order to investigate the potential impact of age on efficacy and safety of catheter ablation in SVT and VT in the very elderly we performed a propensity matched analysis of ablation procedures in patients with AVNRT, AFL, and VT.

## 2 PATIENTS AND METHODS

The study was conducted in accordance with the guidelines of the Declaration of Helsinki. In the present study, we analyzed our single center prospective ablation database for the period from august 2002 to December 2018. We included all patients >80 years (n = 387) who underwent catheter ablation for typical AFL, AVNRT, or VT and performed a matching for age and sex at time of ablation, and ensured that procedures were similarly distributed over the period of data acquisition. Three comparison groups for each arrhythmia were formed with age groups from 40‐60 years, 60‐80 years, and > 80 years of age. The AFL cohort consisted of 753 patients, the AVNRT cohort of 270 patients, and the VT cohort of 168 patients in total. Acute success rates, hospitalization time, and complications were recorded.

### 2.1 Statistical analysis

Continuous data are reported as mean ± SD, and categorical data are reported as percentages. Statistical analysis was performed using GraphPad PRISM 6.0 (San Diego, CA) and the SPSS Statistics, version 20.0 (SPSS, Inc., IL). A *P*‐value <.05 was considered statistically significant.

## 3 RESULTS

A total of 1191 consecutive patients (397 in each age group) with different underlying arrhythmias (typical AFL: 753 patients, AVNRT: 270 pts, VT: 168 pts, Table 1) were included in this analysis. Of the 1191 patients, about two‐thirds were male (n = 756, 64%). While in AVNRT patients 50% of pts were female, and in AFL pts 38%, only 9% of pts undergoing VT ablation were women. While in AVNRT pts, the majority had a normal LV function irrespective of patients' age (92%‐99%), impaired LV function was present in up to 30% of the AFL cohort (>80 years) and in more than 80% of the VT cohort (>80 years). Correspondingly, most VT patients >60 years (90‐96%, Table 1) had structural heart disease, mostly coronary artery disease (CAD) (80%‐82%, Table 1). This was also true for a relevant number of AFL (>80 years/60‐80 years: 36.7%/27.1%, respectively), and AVNRT patients (>80 years/60‐80 years: 21.1%/23.3%, respectively) older than 60 years. Younger patients age suffered notably less often from CAD (age 40‐60 years: AFL 6.8%, AVNRT 3.3%, VT 46.4%) as they also had substantially less risk factors (arterial hypertension, diabetes, end‐stage renal disease). Valvular heart disease and valve replacements were most common in AFL patients (up to 20%) while AVNRT patients and also VT patients had very few valvular disorders (see Table 1).

**TABLE 1:** Patient characteristics at baseline

	Typical AFL (n = 753) (>80y/60‐80y/40‐60y)	AVNRT (n = 270) (>80y/60‐80y/40‐60y)	VT (n = 168) (>80y/60‐80y/40‐60y)
Age (years)	82.5/70.1/53.0	83.0/67.1/49.8	82.4/68.9/52.5
Male (%)	62.2	50.0	91.1
Hypertension (%)	70.9/ 82.9^a^/57.0^a^	60.0/64.4/38.9^a^	78.6/94.6^a^/82.1
Diabetes mellitus (%)	16.7/20.7/8.8^a^	16.7/20.0/7.8	23.2/28.6/16.1
Dialysis‐dependent CKD (%)	2.8/0.8/1.6	2.2/3.3/0.0	8.9/5.4/1.8
Coronary artery disease (%)	36.7/27.1/6.8	21.1/23.3/3.3^a^	82.1/80.4/46.4^a^
Prior MI (%)	9.2/13.1/4.4^a^	8.9/6.7/2.2	41.1/55.4/44.6
Prior CABG (%)	15.1/13.9/2.0^a^	4.4/3.3/0.0	19.6/28.6/14.3
NICM (%)	5.2/8.0/13.9	3.3/1.1/2.2	8.9/16.1/17.9
Prior cardiac device (%)	10.8/12.7/7.2	6.7/2.2/1.1	73.2/71.4/39.3^a^
Severe valvular disease (%)	19.1/13.5/9.2^a^	6.7/3.3/2.2	5.4/5.4/3.6
Prior valve replacement (%)	8.4/10.0/6.8	2.2/0.0/2.2	0.0/1.8/3.6
BMI (kg/m^2^)	26.1/27.0/28.0	25.5/27.0/23.0	25.6/28.0/28.0
LV‐function			
Normal (%) Impaired	72.9/78.5^a^/89.2^a^	92.2 /92.2/98.9	17.9/19.6/44.6^a^
Mild (%)	8.8/10.0/4.0^a^	3.3/4.4/1.1	7.1/14.3/14.3
Moderate (%)	8.4/8.4/4.4	3.3/0.0/0.0	25/16.1/12.5
Severe (%)	8.0/3.1/2.4^a^	1.1/2.2/0.0	50.0/50.0/28.6^a^

*Note*: Reference, age group >80 years for the respective arrhythmia.

Abbreviations: BMI, body mass index; CKD, chronic kidney disease; CABG, coronary artery bypass graft; MI, myocardial infarction; NICM, non‐ischemic cardiomyopathy.

^a^ Significant difference compared to reference (*P* < .05).

BMI was comparable in all age groups and arrhythmias and ranged from 25.5 to 28.0 kg/m^2^. Mean time of hospitalization ranged from 2.5 days for young AVNRT patients to 13.5 days for VT patients older than 80 years (see Table 2).

**TABLE 2:** Results

	Typical AFL (n = 753) (>80y/60‐80y/40‐60y)	AVNRT (n = 270) (>80y/60‐80y/40‐60y)	VT (n = 168) (>80y/60‐80y/40‐60y)
Acute success of ablation (%)	96.4/98.4/98.4	96.7/94.4/96.7	82.1/83.9/92.9
Re‐do procedure (%)	0.8/0.4/0.0	1.1/1.1/0.0	5.4/3.6/3.6
In‐stay complications (%)	2.8/5.6/2.8	4.4/2.2/2.2	16.1/14.3/3.6^a^
Early recurrence	2.8/0.4/0.4	2.2/0.0/1.1	12.5/10.7/1.8^a^
Vascular without OP	0.0/2.0/0.0	2.2/2.2/1.1	0.0/1.8/0.0
Vascular with OP	0.0/0.0 0.4		3.6/1.8/0.0
Pericardial effusion	0.0/0.4/0.4		
Infection	0.0/2.8/1.6		0.0/3.6/1.8
Start with new AAT after ablation (%)	18.7/17.5/17.9	10.0/8.9/6.7	44.6/44.6/25.0^a^
Implantation of cardiac device (ILR, pacemaker, ICD) (%)	10.0/9.6/4.0^a^	5.6/1.1/0.0^a^	5.4/8.9/7.1
Duration of hospital stay (d)	5.6/4.1/3.7	5.4/3.2/2.5	13.5/10.0/5.3

*Note*: Reference, age group >80 years for the respective arrhythmia.

Abbreviations: AAT, antiarrhythmic therapy; AFL, atrial flutter; AVNRT, atrioventricular nodal re‐entry tachycardia; ICD, implantable cardioverter defibrillator; ILR, implantable loop recorder; OP, operation; VT, ventricular tachycardia.

^a^ Significant difference compared to reference (*P* < .05).

Complication rates were low for AFL and AVNRT patients of all age groups (2%‐5%) and higher in VT patients (up to 15%, Table 2), mostly due to early recurrences of arrhythmia which were classified as a complication. There was no death during the ablation procedure. Two patients died from cardiac arrest in cardiogenic shock the day after the VT ablation (one in age group 40‐60 years and one >80 years, respecctively). Another patient died from pulmonary sepsis (VT group >80 years). No patient in the AFL or AVNRT group died during hospitalization/hospital stay (Tables 1 and 2).

## 4 DISCUSSION

In this study, we present data on the acute outcome of catheter ablations of typical AFL, AVNRT, and VT in patients >80 years and younger matched comparison groups. The study cohorts consisted of a rather typical population with different histories of structural heart disease and risk factors (see Table 1). As expected, more patients with with AFL and VT had cardiovascular risk factors compared to patients with AVNRT and prevalence of risk factors increased with age.

In AFL we found high acute success rates and fortunately, in our analysis there were fewer complications than reported in a recent registry data analysis by Steinbeck et al.^4^ No in‐hospital death or major adverse event occurred in our population. However, there was a certain risk for pacemaker implantation after successful ablation. In our data analysis, about 4% to 10% of AFL patients received a cardiac device during the same hospital stay (see Table 2). Similar results could be shown by an observational study from Taiwan.^11^ The most common reason was disturbed AV conduction in sinus rhythm or sick sinus syndrome and intended initiation of an antiarrhythmic therapy for concomitant AF. Although patients aged >80 years or 60‐80 years had considerably more comorbidities (>80 years, 60‐80 years: CAD 36.7%/27.1% vs 6.8% [40‐60 years]; normal LV function 72.9/78.5% vs 89.2%; respectively), ablation success as well as complication rates were comparable in all groups. This underlines that CTI ablation is a procedure which can be performed safely and successfully also in older patients with more comorbidities.

Likewise, high success rates of ablation were also found for slow pathway modulation in AVNRT. While recurrence rates of AVNRT in the pediatric and adolescent population appear to be higher and range from 10%^12^ up to over 20%,^13^ success rates of slow pathway modulation in an adult population are high and well above 95%^14^, ^15^ and therefore in line with results from our trial. One may speculate that the ablation approach is performed more cautiously in children to minimize the risk of AV block and to avoid the necessity of pacemaker implantation. Interestingly, ablation seems to be more complicated in young patients than in older ones. AVNRT ablation is probably one of the most successful procedures with a high immediate and long‐term success and seems to work equally efficient in older patients. This is in line with recent data from the German Ablation Registry.^16^ In our study, there was no difference concerning neither ablation success nor complication rates between the different age groups, although we have to underline that the youngest patients were 40 years of age and therefore not comparable to the adolescent populations mentioned earlier. The higher rate of device implantation (>80 years: 5.6% vs <60 years: 0%) was not driven by AV block as a result of slow pathway modulation. There was no iatrogenic AV block in our AVNRT cohorts. In fact, cardiac devices were only implanted because of other arrhythmias being diagnosed before or during hospitalization. Hence, in case of symptomatic tachycardia and either electrophysiological proof of dual AV nodal anatomy or ECG documentary of AVNRT, ablation should be recommended as standard therapy of AVNRT therapy even in the very elderly.

The more challenging VT ablations were also performed with a high acute success rate of over 80% in patients aged >80 years. Short time recurrence rate of VT was quite high with up to 12.5%. A total of 3.6% to 5.4% of patients underwent a reablation during the same hospital stay. Moreover, almost half of the patients aged over 60 years received additional antiarrhythmic drug therapy during the stay. Frontera et al^17^ presented similar results of VT ablation in octogenarians. They demonstrated a good safety profile and a relatively high mid‐term survival of these patients compared to a younger control group. Our results support the finding, that VT ablation can be performed with a good safety and benefit profile also in the very elderly. Comparing the three age cohorts, it becomes obvious that people younger than 60 years essentially differ from the older patients from 60 to 80 years or even over 80 years. While 80% of the VT patients >60 years suffered from underlying CAD and already had an ICD in >70%, in the patients aged <60 years CAD was prevalent in only 46% and only 39% of patients had an ICD. In addition, ablation success was very high for a VT cohort in patients <60 years (92.9%), complications rates were lower and the duration of hospital stay was half as long. This was due to the fact that about a third of VT (36%) were of idiopathic nature in these patients as compared to <10% in the two older age groups. This limits the comparability of the three age groups but illustrates the fact that a benign idiopathic nature of VT becomes more unlikely with increasing age.

A dutch working group underlined a different decisive factor for the outcome of VT ablation rather than age—namely the high impact of LV dysfunction in a study of patients with ischemic cardiomyopathy.^18^ Nevertheless, higher age was associated with worse outcome in two contemporary risk scores predicting survival and recurrence risk in patients undergoing VT ablation.^19^, ^20^ As VT ablation is often not an elective procedure and as the reduction of events has direct impact on patients' outcome and prognosis, there is no alternative option in most cases. Consequently, data on these procedures are also of eminent importance to optimize procedure and success rates.

## 5 CONCLUSION

Our data underline that various catheter ablation of AFL, AVNRT, and VT worked effectively and with low complication rates in this very elderly. Ablation of AFL as well as AVNRT were performed with very high acute success rates of >95% across all age groups although patients' comorbidities were clearly increasing with age. VT ablation also had a good acute success rate of over 80% in patients >60 while success rates were even higher in VT patients <60 years because of age‐dependent decrease of idiopathic VT.

### 5.1 Limitation

This study has several limitations, mostly associated to the retrospective nature of the data. Patients with AF were not included in our analysis as only a limited number of patients over 80 years underwent pulmonary vein isolation at our institution between August 2002 and December 2018. Furthermore, although propensity matching was carefully performed, there is always a risk for a certain selection bias and confounding factors.

## CONFLICT OF INTEREST

The authors declare no potential conflict of interest.

## Data Availability

The data that support the findings of this study are available from the corresponding author upon reasonable request.
